# I Approach Therefore I am? Isolating Behaviour-Specific Effects on Self-Evaluation

**DOI:** 10.5334/irsp.1053

**Published:** 2026-07-13

**Authors:** Ivane Nuel, Marie-Pierre Fayant, Theodore Alexopoulos

**Affiliations:** 1 Centre de Recherches sur la Cognition et l’Apprentissage, Université de Poitiers, Université de Tours, CNRS, Poitiers, France; 2 Laboratoire de Psychologie Sociale, Université Paris Cité Boulogne-Billancourt, 92100 Paris, France; 3 Laboratoire de Psychologie, UR 4239, University of Bordeaux, Bordeaux, France

**Keywords:** approach-avoidance, assimilation, contrast, self-construal, self-evaluation

## Abstract

Research suggests that approach and avoidance—decreasing or increasing self–stimulus distance, respectively—influence self-evaluation. However, this conclusion typically stems from examining approach and avoidance as a pair, while no empirical research has satisfactorily isolated the unique causal contribution of each behaviour. To address this gap, three experiments (*N*_Total_ = 1929) used an adapted Manikin Task incorporating a proper control condition (i.e., no distance change) to first isolate the effect of approach, relying on both reaction time and self-report measures. Furthermore, by incorporating avoidance in the last experiment, this task allowed us to oppose model-based theoretical predictions. Results did not reveal any behaviour-specific effects. Nevertheless, the proposed methodology opens new avenues for investigating the behavioural determinants of self-construal.

How individuals define and evaluate themselves is central to psychological functioning and drives the core processes of the self (Markus & Wurf, 1987; Sedikides & Strube, 1997). This self is shaped by social and situational interactions (Morf & Mischel, 2012), and research suggests that interactions involving changes in distance between the self and a stimulus in the social environment influence self-construal. We argue that this literature would greatly benefit from a methodological refinement that enables the isolation of specific behavioural effects in this self-construal. Here, we take a step in that direction by proposing a task designed to make this possible.

## Self-Construal as Person–Environment Interactions

Individuals acquire self-knowledge through social/situational interactions and exposure to available social information. Encountered information can shape self-construal through self-assimilative or self-contrastive effects; that is, self-evaluations are displaced toward or away from contextual features (Suls & Wheeler, 2007). This produces either a positive or negative correlation between self-evaluations and contextual features, respectively. For example, in highly skilled peer contexts, self-assimilation (or contrast) refers to an increase (or decrease) of perceived competence/performance. Which effect occurs is conditional on the context, including the specific way individuals engage with their environment. To interact with their surroundings, individuals are generally energised to approach or avoid (Brendl & Higgins, 1996; Elliot, 2006). Namely, they (physically) reduce or increase the distance between themselves and a stimulus (Solzbacher et al., 2025; van Dantzig et al., 2008). In general, research suggests that approach and avoidance behaviours lead self-evaluations to become, respectively, positively correlated (assimilated) and negatively correlated (contrasted) with contextual features.^1^

A handful of studies corroborate the self-evaluative effects of approach and avoidance (Batailler et al., 2021; Boissicat et al., 2022; Fayant et al., 2011; Kawakami et al., 2008; Nussinson et al., 2010; Phills et al., 2011). Most of them use an approach-avoidance training paradigm: Individuals repeatedly approach (vs. execute another movement toward) social information (e.g., a social group, but see Boissicat et al., 2022 and Nussinson et al., 2010). Then, self-evaluation is assessed via a self-Implicit Association Test (IAT, Greenwald et al., 1998) or self-reports. For instance, Phills and colleagues (2011) asked participants to repeatedly approach (avoid) outgroup members by pulling (pushing) a joystick toward (away from) the self, or moving a circle representing the self toward (away from) outgroup members. This approach (vs. avoidance) training increases the IAT score supposed to capture self-associations with the outgroup (see also Kawakami et al., 2008). Fayant and colleagues (2011) showed that participants evaluate themselves as more attractive after having repeatedly walked toward the picture of an attractive (vs. moderately attractive) person, an assimilation effect, whereas no such effect was observed in the avoidance condition.

Given the functional role of approach and avoidance—defined as reducing or increasing physical distance between the self (or its representation) and a stimulus, respectively (Solzbacher, Koenig, & Walter, 2025; van Dantzig, Pecher, & Zwaan, 2008)—it is important to understand how they shape self-construal. Yet, so far, no empirical work has isolated the causal contribution of these behaviours, due primarily to specific methodological choices and limitations. Addressing this gap is crucial for understanding the effects of individuals’ behaviours in self-construction, their underlying mechanisms, and ultimately for testing competing theoretical explanations. Accordingly, we propose a relevant methodology designed to isolate the causal effects of specific behaviours on self-evaluation. Here, we focus first on the self-assimilative effect of approach, as individuals initially engage with stimuli in their environment by approaching them, particularly during an exploratory phase, before determining that avoidance is necessary. Subsequently, we also consider avoidance to capitalise on this methodology for testing competing theories.

## Singling Out the Effect of Approach on Self-Construal: Outline of a Strategy

Isolating the influence of approach–avoidance behaviours requires some precautions. Here, we illustrate these precautions in the process of isolating the effect of approach. First, testing causality hinges on an appropriate control condition that cannot be reinterpreted as avoidance, otherwise the source of the effect remains unclear. For instance, even some appealing control conditions based on lateral movements (Phills et al., 2011) or ‘no action’ (Nussinson et al., 2010) do not satisfy this criterion. Lateral movements increase the self-stimulus distance and no action/freezing are defensive reactions to threat (Corr & McNaughton, 2012). Besides providing a clear-cut interpretation, a control condition should also address confounds related to the amount of visual information and action involved. As one approaches an object, the amount of collectable information about this object increases. In addition, comparing approach behaviours to a ‘no action’ condition involves comparing various action quantities. The ‘action’ condition could activate more strongly the self by providing richer sensorimotor information (Gallagher, 2000), or by orienting attention toward the moving self-related stimulus (Franconeri & Simons, 2003). Thus, compared with a ‘no action’ condition, the approach condition may yield self-evaluations more aligned with the social stimulus, not because of distance reduction per se, but because it provides more information about the stimulus or the self. It is thus crucial to develop a control condition that cannot be conceptualised as avoidance, while addressing these confounds.

Second, isolating the effect of approach requires ruling out alternative interpretations of the targeted behaviours. Yet, the same movement can signal approach or avoidance depending on whether participants use the self or the object as a reference point (Seibt et al., 2008). Finally, because many approach–avoidance behaviours in everyday life are not assumed to stem from explicit external instructions, it is crucial to carefully control for instructional effects in experiments. If behaviour-specific effects on self-evaluation require explicit instructions to occur, concerns about demand bias resurface (Corneille & Béna, 2023). Instructions may influence the self through propositional reasoning (e.g., instructions to approach stimuli may produce the inference of self-similarity; Van Dessel et al., 2017) without any behaviour being performed. Although propositional reasoning represents a viable explanation of approach–avoidance effects on self-evaluation, such reasoning need not rely on explicit instructions, as propositions may also arise from behaviour itself. However, if the relevant propositions stem primarily from instructions, the observed effect may be driven by instructions rather than by the behavioural manipulation, thereby undermining internal validity.

So far, no research has directly tackled the aforementioned issues. Previous attempts do not clearly isolate the contribution of approach—largely due to the absence of a proper control condition, which was not their primary aim (Boissicat et al., 2022; Fayant et al., 2011; Kawakami et al., 2008; Nussinson et al., 2010; Phills et al., 2011). To address this issue, we aim to move beyond previous paradigms and isolate the influence of specific behaviours (e.g., approach) on self-construal.

## Overview

We argue that a better understanding of the behavioural determinants of self-construction processes would benefit from a methodology designed to isolate the causal effect of specific behaviours. To enable this, we propose an adapted manikin task (MT, De Houwer et al., 2001), which presents several advantages. The MT has been used extensively in the literature to investigate approach–avoidance via a third-person perspective (e.g., Hütter & Genschow, 2020; Van Dessel et al., 2018). In our version, participants have to categorise words as adjectives (from a specific dimension) or common nouns. The contingency between categorisation and specific behaviours is manipulated. When participants correctly categorised an adjective, a virtual figure representing the self either: 1) moved toward the stimulus (approach), 2) performed a movement that did not change the self-stimulus distance (e.g., an upward arm movement or assuming a seated position; control), or 3) moved away from the stimulus (avoidance, Experiment 3). Task instructions focused on word categorisation, not on the movements of the figure.

This MT isolates the effect of approach, which is the focus of this initial stage of the research: First, it relies on a proper control condition hardly interpretable as avoidance. Indeed, the control behaviour does not entail any distance change and involves a comparable amount of action as approach. As the MT adopts a third-person perspective, it also controls adequately for the amount of visual information. Second, the MT avoids any interpretational ambiguity concerning the target behaviours by forcing participants to adopt a self-reference point (Krieglmeyer & Deutsch, 2010). Finally, this task minimises demand biases and the instruction effect. Instructions target irrelevant stimulus features (e.g., grammatical nature) and deemphasise the focal behaviour–valence contingency.

Using this task, we predicted across all three experiments that approach increases self-assimilation. Furthermore, by isolating specific behavioural effects, and by including avoidance, this MT allowed us to oppose model-based theoretical predictions in the final experiment.

We collected and analysed data anonymously in accordance with the American Psychological Association’s ethical principles. We did not seek ethics approval as it was not required under applicable national regulations. Supplementary Material is available in the Open Science Framework (OSF): https://doi.org/10.17605/OSF.IO/CFRJ2.

**Figure 1:**
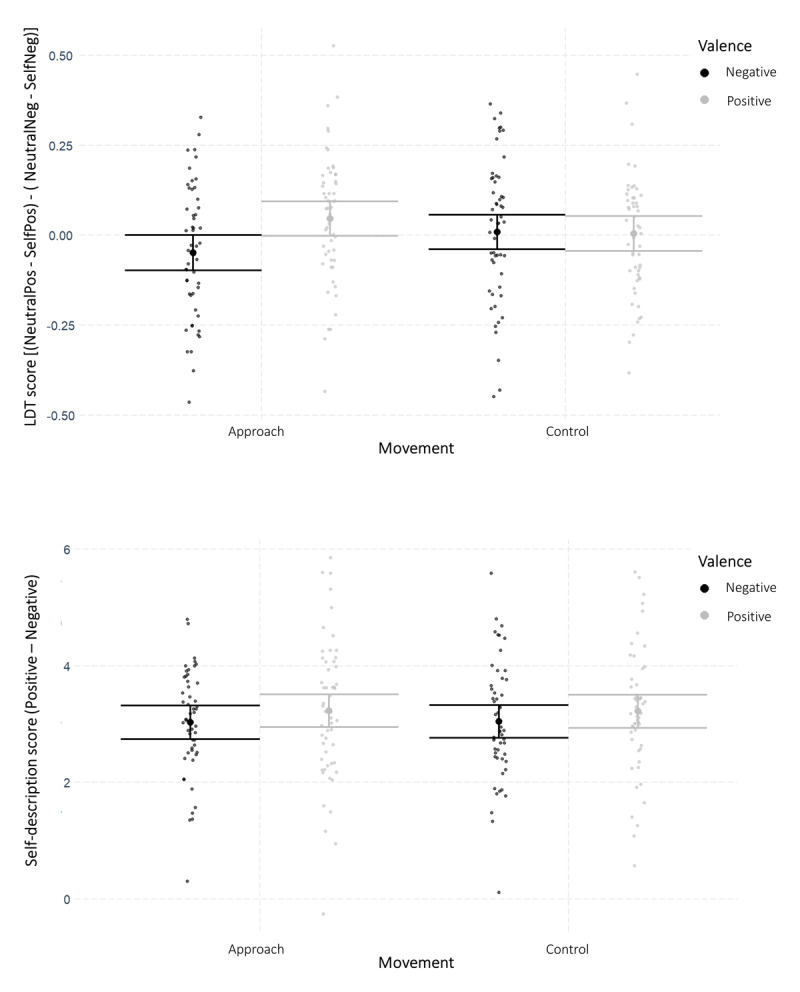
Self-evaluation proxies depending on stimuli valence and movement in Experiment 1 (Error bars represent 95% confidence intervals).

The 90% confidence intervals (CIs) reported hereafter are based on the partial eta-squared.^2^ We reported both frequentist and Bayesian analyses—using the Jeffreys-Zellner-Siow default Bayes Factor (BF; calculated using the *BayesFactor* R package, version 0.9.12-4.7; Morey et al., 2024)—with the *BF*_10_ indicating evidence in favour of H_1_ as compared to H_0_. *BF*s interpretations are based on Lee and Wagenmakers’ (2013) classification. All results are reported in Table 1 and graphs in Figure 1. In the main text, we only report results testing the self-evaluative effect of behaviour.

**Table 1:** Results for Experiments 1, 2, and 3.


EXPERIMENT 1									

**LDT score**	**M (SD)**				**Effect**	**Test**	***PRE* [90% CI]**	***p***	**BF_10_**

		**Control**	**Approach**		***Training valence***	*F*(1, 208) = 3.35	.02 [.00, .05]	.069	1.21

	**Negative**	0.009 (0.18)	–0.05 (0.19)	–0.02 (0.19)	***Contingent movement***	*F*(l, 208) = 0.11	.00 [ 00, .005]	.74	0.27

	**Positive**	0.004 (0.17)	0.05 (0.17)	0,03 (0,17)	***Interaction***	*F*(1, 208) = 4,07	.03 [.01, .08]	.045	1.69

		0.01 (0.17)	–0.00 (0.19)						

**Self-description task**	**M (SD)**				**Effect**	**Test**	***PRE* [90% CI]**	***p***	**BF_10_**

		**Control**	**Approach**		***Training valence***	*F*(l, 208) = 1.71	.01 [.00, 04]	.192	0.64

	**Negative**	3.04 (1.00)	3.03 (0.89)	3.04 (0.94)	***Contingent movement***	*F*(l, 208) = 0.00	.00 [.00, .00]	.99	0.28

	**Positive**	3.22 (1.07)	3.23 (1.20)	3.22 (1.13)	***Interaction***	*F*(l, 208) = 0.01	.00 [.00, 00]	.923	0.28

		3.13 (1.04)	3.13 (1.06)						

**EXPERIMENT 2**									

**IAT score**	**M (SD)**				**Effect**	**Test**	***PRE* [90% CI]**	***p***	**BF_10_**

		**Control**	**Approach**		***Training valence***	*F*(l, 213) = 0.16	.00 [.00, .02]	.686	0.29

	**Negative**	0.34 (0.52)	0.22 (0.52)	0.28 (0.52)	***Contingent movement***	*F*(l, 213) = 0.14	.00 [ 00, .02]	.709	0.29

	**Positive**	0.27 (0.42)	0.35 (0.54)	0.31 (0.48)	***Interaction***	*F*(l, 213) = 2.23	.01 [.00, .04]	.137	0.76

		0.30 (0.47)	0.28 (0.53)						

**Self-feeling thermometer**	**M (SD)**				**Effect**	**Test**	***PRE* [90% CI]**	***p***	**BF_10_**

		**Control**	**Approach**		***Training valence***	*F*(l, 213) = 0.00	.00 [.00, .00]	.99	0.27

	**Negative**	66.04 (16.30)	62.77 (21.36)	64.38 (19.03)	***Contingent movement***	*F*(l, 213) = 0.00	.00 [.00, .00]	.956	0.27

	**Positive**	62.67 (18.07)	66.21 (17.11)	64.33 (17.64)	***Interaction***	*F*(l, 213) = 1.87	.01 [.00, .04]	.173	0.64

		64.22 (17.28)	64.49 (19.34)						

**Signature size**	**M (SD)**				**Effect**	**Test**	***PRE* [90% CI]**	***p***	**BF_10_**

		**Control**	**Approach**		***Training valence***	*F*(l, 211) = 0.00	.00 [.00, 00]	.953	0.28

	**Negative**	–0.10 (1.84)	0.31 (1.77)	0.11 (1.81)	***Contingent movement***	*F*(l, 211) = 0.37	.00 [.00, .02]	.543	0.33

	**Positive**	0.18 (1.53)	0.06 (1.73)	0.13 (1.62)	***Interaction***	*F*(l, 211) = 1.26	.01 [.00, .03]	.264	0.49

		0.06 (1.68)	0.19(1.75)						

**EXPERIMENT 3**									

**IAT score**	**M (SD)**				**Effect**	**Test**	***PRE* [90% CI]**	***p***	**BF_10_**

	**Avoidance**	**Control**	**Approach**		***Approach vs. Control***	*F*(l, 1497) = 0.73	.00 [.00, .00]	.394	0.13

	0.48 (0.37)	0.51 (0.36)	0.53 (0.37)		***Avoidance vs. Control***	*F*(1, 1497) = 0.95	.00 [.00, .00]	.330	0.14

**BFI score**	**M (SD)**				**Effect**	**Test**	***PRE* [90%CI]**	***P***	**BF_10_**

	**Avoidance**	**Control**	**Approach**		***Approach vs. Control***	*F*(1, 1497) = 0.52	.00 [.00, .00]	.470	0.12

	0.80 (0.73)	0.80 (0.69)	0.83 (0.69)		***Avoidance vs. Control***	*F*(l, 1497) = 0.01	.00 [.00, .00]	.903	0.09


## Experiments 1 and 2

In Experiment 1, we relied on a modified lexical decision task (LDT; Dijksterhuis et al., 1998) and a self-description task (Sanitioso & Niedenthal, 2006). In Experiment 2, because the IAT is usually used (Batailler et al., 2021; Kawakami et al., 2008; Phills et al., 2011; Van Dessel et al., 2017), but is also subject to criticisms (e.g., recoding strategies, relativity of the measure), we relied on two unipolar Single Category IATs in a recoding-free version^3^ (RF-SC-IAT; Haynes et al., 2016; Lebens et al., 2011). We also used a self-feeling thermometer and signature size changes as proxies of self-evaluation (Zweigenhaft & Marlowe, 1973). Although these experiments were not pre-registered, we remained fully transparent and reported sample size determination, all data exclusions, manipulations, and measures. Of note, the measurements proved to be unreliable, so the results should be interpreted with caution.

### Sample Size Determination

By comparing approach to avoidance, previous research complicates the estimation of the effect size of approach. Concerning reaction-time (RT) based measures, effects range from *d* = 0.51 to *d* = 1.02 (Phills et al., 2011). Concerning self-reports, the only effect sizes we are aware of in the literature are those of Fayant and colleagues (2011) ranging from *d* = 0.37 to *d* = 0.50.^4^ However, even the smallest of these effects could potentially overestimate the specific effect of approach as avoidance may also contribute to the observed effect. Thus, for both experiments we planned to run at least 50 participants per condition following the prevailing recommendations at that time (Simmons et al., 2013). We adjusted the alpha-level to the number of measures in each experiment. Given our settings, such a criterion enabled us to detect an effect size *d* = 0.44 (η^2^ = .047) with a power of 80% and an adjusted alpha-level of .025 for Experiment 1 and an effect size *d* = 0.46 (η^2^ = .051) with a power of 80% and an adjusted alpha-level of .017 for Experiment 2.

### Method

#### Participants

French-speaking undergraduate psychology students participated in the study voluntarily or in exchange for partial course credits (*N*_Experiment1_ = 217; *N*_Experiment2_ = 226). They were randomly assigned to a 2 (training valence: positive, negative) × 2 (contingent movement: approach, control) between-participants design. We excluded participants because of self-reported dyslexia (*n*_Experiment1_ = 2; *n*_Experiment2_ = 1), technical issues (*n*_Experiment1_ = 1; *n*_Experiment2_ = 2), missing data (*n*_Experiment1_ = 1) or scoring above four (Judd et al., 2009) on studentised deleted residuals (*n*_Experiment1_ = 1; *n*_Experiment2_ = 1). In Experiment 2, we also excluded two participants due to a misspelling of their first name, and three others that produced more than 10% of RTs below 300 ms (Greenwald et al., 2003).^5^ Our final sample included 212 participants in Experiment 1 (171 women, *M_Age_* = 19.89, *SD_Age_* = 2.20, missing age data for 45 participants due to hasty spacebar keypresses) and 217 participants (148 women, *M_Age_* = 21.81, *SD_Age_* = 4.26) in Experiment 2.

#### Procedure

**Approach Training**. Participants first signed a consent form. In Experiment 2, we also collected self-related stimuli (i.e., first and last names, close other names) for the RF-SC-IATs and asked participants to sign a pseudo-form for the baseline signature size. Then, participants performed the MT. They were informed that words will appear on screen, accompanied by a figure representing the self. To facilitate identification with the figure, we constantly labelled it with participants’ first names. Participants were instructed to press one key if the word was an adjective and another key if the word was a common noun. Each categorisation triggered a movement, and participants pressed the correct key four consecutive times, as quickly as possible, until movement completion. Initially, the figure’s movement was unspecified, and it was during the task that participants learned that it could either approach the words or move on the spot.

Participants underwent two blocks, including six training trials followed by 96 experimental trials. In a block, 12 valenced adjectives (Ric et al., 2013) and 12 neutral common nouns (Bonin et al., 2003) were presented at the centre of the screen: twice with the figure appearing on the left and twice with the figure appearing on the right. All common nouns were neutral and the valence of the adjectives was contingent on the condition. In the positive condition, adjectives were positive personality traits, whereas in the negative condition adjectives were negative personality traits. In the approach condition, when participants correctly categorised an adjective, the virtual figure moved toward the stimulus (i.e., an approach movement). When they correctly categorised a noun, the figure lifted its arm upwards (i.e., a control movement). These contingencies were reversed in the control condition. Consequently, the amount of action was comparable across conditions and the only manipulated feature was the approach-valence contingency. For incorrect responses, error feedback terminated the trial. Response-key assignment was counterbalanced across blocks, and the order of blocks across participants. Then, proxies of self-evaluation were assessed via different tasks. In the end, participants indicated their gender, age and French language skill.^6^

In Experiment 1, after the MT, participants performed a primed LDT in which they had to determine whether stimuli (i.e., positive or negative personality traits, neutral words and nonwords) are legal French words (Dijksterhuis et al., 1998). Half of the stimuli were preceded by self-related primes and the other half by self-unrelated primes. In the LDT, priming self-related stimuli should activate participants’ self-evaluation, which should influence RT for categorising positive and negative words. The difference in RT for categorising positive and negative words primed by self-related stimuli is compared with the difference in RT for categorising positive and negative words primed by non-self-related stimuli (Spalding & Hardin, 1999). Finally, participants indicated on a 6-point scale from 1 (*not at all*) to 6 (*very much so*) to what extent 21 traits, different from the previous tasks, described themselves at this very moment (i.e., 8 positive, 8 negative and 5 neutral fillers; Sanitioso & Niedenthal, 2006).

In Experiment 2, after the MT, participants performed a positive and a negative RF-SC-IAT. In both RF-SC-IATs, the critical block involved both categorisation of valenced (positive or negative depending on the IAT) and neutral words and categorisation of self-related words. The possible configurations always opposed combined vs. single labels and involved either: self and valenced category assigned to the same key (combined labels) vs. neutral category to the opposite key (single label), or self and neutral category assigned to the same key vs. valenced category to the opposite one. These label configurations and stimuli presentations were randomised. While response assignment for valence categorisation remained constant throughout the task, key assignment for the ‘self’ category randomly switched from left to right between trials. The order of the two IATs was counterbalanced across participants. Finally, participants evaluated their opinion of themselves (on a scale from 0 to 100) and signed a second pseudo-form (signature size after treatment).

### Results

#### Data Preparation

In Experiment 1, the LDT showed a very low split-half reliability (*r* = .02; computed on odd-even trials). Response times (RTs) were trimmed according to Tukey’s criterion^7^ (Tukey, 1977; excluding 6.7% of all trials) and log-transformed to correct for positive skew. We excluded one word (‘poltroon’) from the analysis as it was unfamiliar to 24.3% of our participants and was an outlier on accuracy (60%). We computed an LDT self-evaluation score by subtracting RT*_Positive Traits_* from RT*_Negative Traits_* for self-related and self-unrelated (neutral) trials separately. We then computed the difference between these scores such that higher values indicated more positive self-evaluations (Spalding & Hardin, 1999). For the self-description task, we computed a self-evaluation score by subtracting the average score on the eight negative traits (α = .63) from the average score on the eight positive ones (α = .73).^8^ Higher composite scores indicated a more positive self-evaluation.

In Experiment 2, the RF-SC-IATs also have low split-half reliability (computed on odd-even trials; *r*_Positive_ _IAT_ = .21; *r*_N__egative IAT_ = .07). For each unipolar RF-SC-IAT, we calculated a score using the improved D-algorithm (Greenwald et al., 2003) such that higher positive (negative) D-scores implied more positive (negative) self-evaluations. From these two scores, we computed an index of self-evaluation by subtracting the negative D-score from the positive D-score such that a positive difference reflected a more positive self-evaluation.^9^ For the signature size proxy, the width × length product was used as a measure of the total area covered by the signature before and after the training (Zweigenhaft & Marlowe, 1973). We then subtracted the first signature size to the second so that a positive number indicated an increase in signature size.

#### Data Analyses

**Figure 2:**
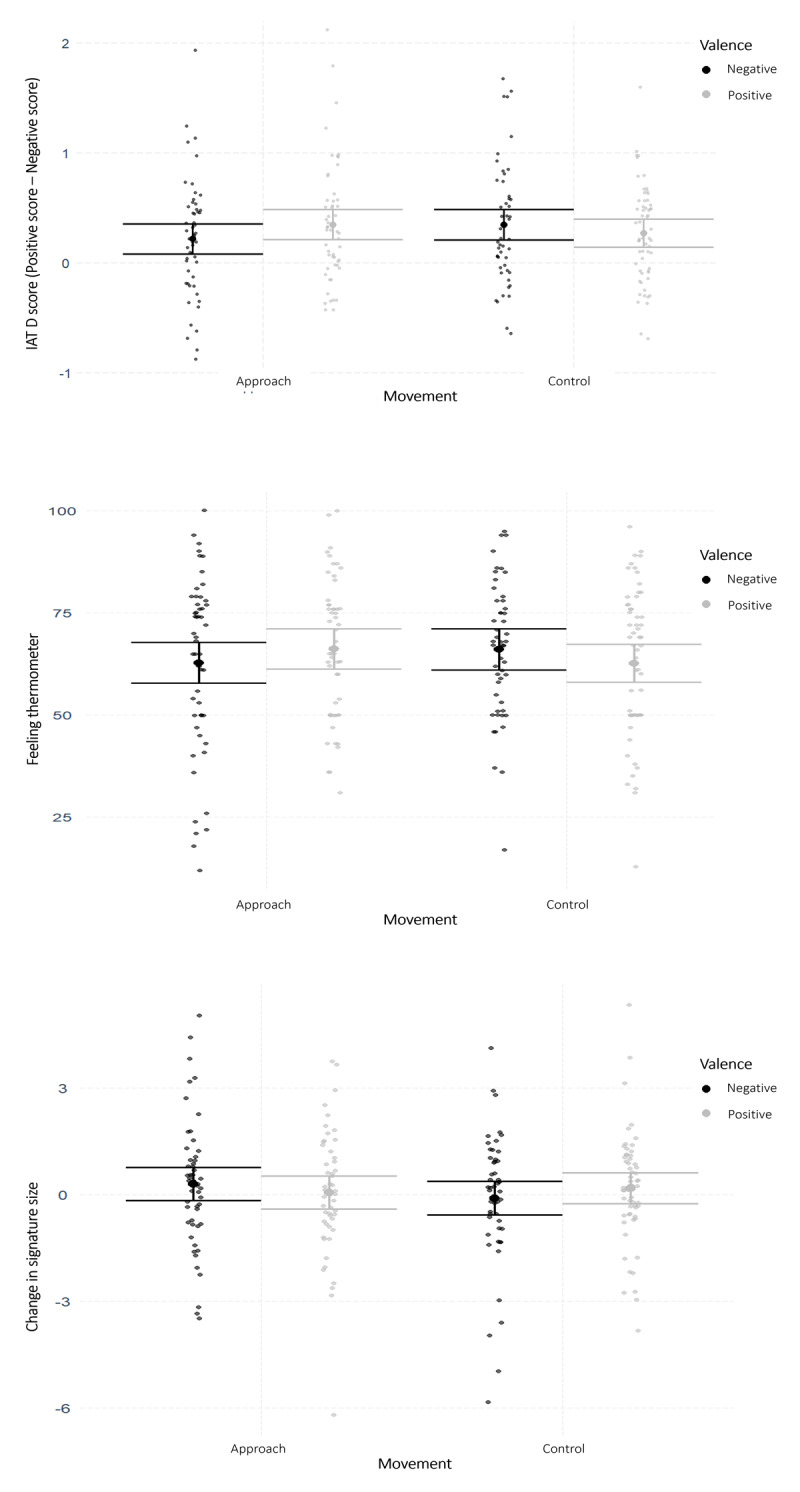
Self-evaluation proxies depending on stimuli valence and movement in Experiment 2 (Error bars represent 95% confidence intervals).

For each experiment, we submitted self-evaluation scores to a 2 (training valence: positive, negative) × 2 (contingent movement: approach, control) between-participants analysis of variance. In all analyses, the predicted interaction between training valence and movement did not reach the adjusted alpha threshold (see Table 1, Figure 1, and Figure 2). *BF*s provide anecdotal evidence in favour of the interaction hypothesis for the LDT score, but moderate to anecdotal evidence in favour of the null hypothesis for the other measures.^10^

### Discussion

In Experiments 1 and 2, an adapted MT specifically designed to isolate behaviour-specific outcomes did not reveal an effect of approach. However, these experiments have limitations. First, a rule of thumb is not optimal to determine sample size and the effect might be smaller than what was detectable. Second, participants may have interpreted the control movement (the lifted arm) as approach: Even if start and end self-stimulus distances were equal, the arm passed closer to the stimulus mid-movement. If so, the contingency manipulation may have been less effective to detect an effect. A post-test (*N* = 67) indicated that the arm lift was less perceived as indicative of approach than the approach movement, but still somewhat so (above the centre of the scale *neither avoidance, nor approach*; see Supplementary Material, 3). Third, we used different measures for self-assimilation than previous research, which were not always reliable (low split half-reliability).

Beyond the null results, limitations of the task itself may be raised. Although the crucial manipulation concerned movement-stimuli contingency, participants performed both approach and control movements, which may have influenced their interpretation. Specifically, the control movement may be perceived as avoidance when contrasted to approach. Although the post-test did not favour such a conclusion, this objection remains legitimate. Moreover, without an avoidance condition, we cannot determine whether approach impacts self-evaluation, whether prior effects were driven by avoidance, or whether the effect of self-performed behaviours failed to replicate.

## Experiment 3

In Experiment 3, we extended the methodology to isolate the effect of a second specific behaviour (avoidance) on self-evaluation, demonstrating the paradigm’s utility to theory testing. We increased the sample size based on a priori power analysis. To prevent participants from interpreting the control movement as approach/avoidance, we 1) relied on a different and pretested control movement in which the figure assumes a seated position,^11^ and 2) constrained movement interpretation by initially presenting all movements. We also added an avoidance condition. Finally, because the standard IAT is the most frequently used task in previous literature (Batailler et al., 2021, Kawakami et al., 2008, Phills et al., 2011, Van Dessel et al., 2017), and is generally more reliable (Greenwald et al., 2022), we chose this task. However, we are aware of the criticism concerning the IAT’s reliability and validity and also included a self-report measure of self-evaluation.^12^ Though more reliable than in Experiments 1 and 2, the IAT used in Experiment 3 still contains substantial measurement error. To simplify the design, we focused on a specific dimension in the MT (rather than overall valence), however the task itself remained unchanged and consisted of the grammatical categorisation of adjectives versus common nouns. We chose conscientiousness because it was easier to generate adjectives unconfounded by valence (e.g., responsible, disciplined), which might otherwise have facilitated the task in certain conditions (e.g., approaching positive adjectives).

Including avoidance further illustrated how a task with an appropriate control condition is also theoretically useful. Different explanations account for the self-assimilative effect of approach. According to an inferential explanation, granting that approach is tightly linked to perceptual nearness (Hommel et al., 2001) and signals similarity (Hughes et al., 2018), when individuals approach social stimuli they may form the propositional reasoning ‘I approach what is similar to myself; therefore approached stimuli are similar to me’ (Van Dessel et al., 2017, 2019). According to a self-anchoring explanation, approaching social stimuli involves a distance reduction between the representations of the self and the stimuli (Gawronski et al., 2007) producing a mental association between these representations. If both explanations predict a behavioural effect on self-evaluation, associative self-anchoring predicts an effect only for approach. In line with associative theories, distance reduction during approach should facilitate self–stimulus associations. In contrast, distance increase during avoidance should not produce *non-*self–stimulus associations, as associative processes are not assumed to support negations (Gawronski & Bodenhausen, 2011).^13^ Conversely, the propositional explanation predicts both assimilation in approach and contrast in avoidance, as these behaviours produce two distinct propositions (i.e., ‘approached stimuli are similar to me’, ‘avoided stimuli are different from me’).

### Sample Size Determination

As Experiments 1 and 2 yielded small effect sizes, we conducted a simulations-based power analysis to detect such effects. Because the main goal was to isolate an effect, we compared each distance-change condition with the control condition. Moreover, while a self-anchoring explanation predicts only an approach effect on self-evaluation, a propositional explanation predicts an effect of both approach and avoidance. Accordingly, we simulated data for these two possible patterns and applied a dummy-coded linear regression (see Results). Because two dependent variables were used, we halved the alpha level (α = .025). Five-hundred participants per condition (*N*_Total_ = 1500) enabled us to detect a small effect size (*d* = 0.20) for the two dummy-coded variables with 80% of power and an alpha level of .025 (see the OSF file for details).

### Method

#### Participants

We recruited 1650 participants via Prolific (https://www.prolific.com) who each were compensated £2.50. We excluded participants who failed at least one attentional check (i.e., not responding ‘baguette’ [*n* = 94], ‘roulette’ [*n* = 81], or ‘strongly agree’ when explicitly instructed, [*n* = 27]) or indicated that their data are not reliable (*n* = 7). Following the D2-algorithm of Greenwald et al. (2003), we excluded 47 participants who had more than 10% of RT below 300 ms. No outliers were identified based on studentised residuals, Cook’s distance, or leverage. We analysed the data of the remaining 1500 participants.

#### Procedure

**Approach Training**. Participants indicated their first name and performed the MT. Participants categorised words depending on their grammatical nature (i.e., adjective vs. common noun). They were informed that a figure representing themselves (labelled with their first-name) will move depending on their responses, and were familiarised with these movements. Specifically, a figure appeared next to a fixation cross and participants pressed a key repeatedly to help it complete its movement. They repeated this three times, once for each movement (i.e., approach, sitting, avoidance) in random order. They were then randomly assigned to either the approach, the control, or the avoidance condition. Although only one movement was used in the main task, familiarisation emphasised the distinction between the approach, avoidance, and control movements.

In the MT, participants performed two blocks of 96 trials. In each block, 12 adjectives related to conscientiousness (Boies et al., 2001) and 12 neutral common nouns (from Experiment 2) were presented at the centre of the screen: twice with the figure appearing on the left and twice on the right. In all three conditions, correct noun categorisations triggered a ‘loading’ symbol on the figure. Correct adjective categorisations triggered movement of the figure depending on condition. In the approach condition, the figure moved toward the stimulus (i.e., approaching conscientiousness-related words); in the control condition the figure sat down; and in the avoidance condition, the figure moved away from the stimulus. Participants pressed the correct key five consecutive times until the ‘loading’ symbol or movement was complete. For incorrect responses, error feedback prompted the correct answer. Left and right response-key assignment was counterbalanced across blocks; block order was counterbalanced across participants. After the MT, participants completed an attention check.

**Proxies of Self-evaluation**.^14^ Two measures served as proxies for conscientiousness self-evaluation: a standard IAT (Greenwald et al., 1998) and the items of the Big Five Inventory-2 (BFI-2; Soto & John, 2017; French version of Lignier et al., 2023). In the IAT, the critical blocks (80 trials each) involve both the categorisation of self-related (5) and non self-related (5) words and the categorisation of conscientiousness-related (5) and non-conscientiousness-related (5) words. Between blocks, key assignment was reversed for the conscientiousness categorisation. Key assignment for the self-categorisation task was counterbalanced across participants as well as the order of key assignment for the dimension-categorisation task. After the IAT, participants completed another attention check.

For the 12 conscientiousness items in the BFI-2 (Lignier et al., 2023; Soto & John, 2017), participants indicated their agreement with affirmations about themselves (e.g., I am someone who tends to be disorganised) on a 5-point scale ranging from *Strongly disagree* to *Strongly agree*. Then, participants completed the last attention check. The order of IAT and BFI-2 were counterbalanced across participants.

Finally, participants interpreted the figure movement (on a 6-point scale ranging from *Clearly avoidance* to *Clearly approach*), indicated their age, gender, their answers’ reliability, and were debriefed.

### Results

**Figure 3:**
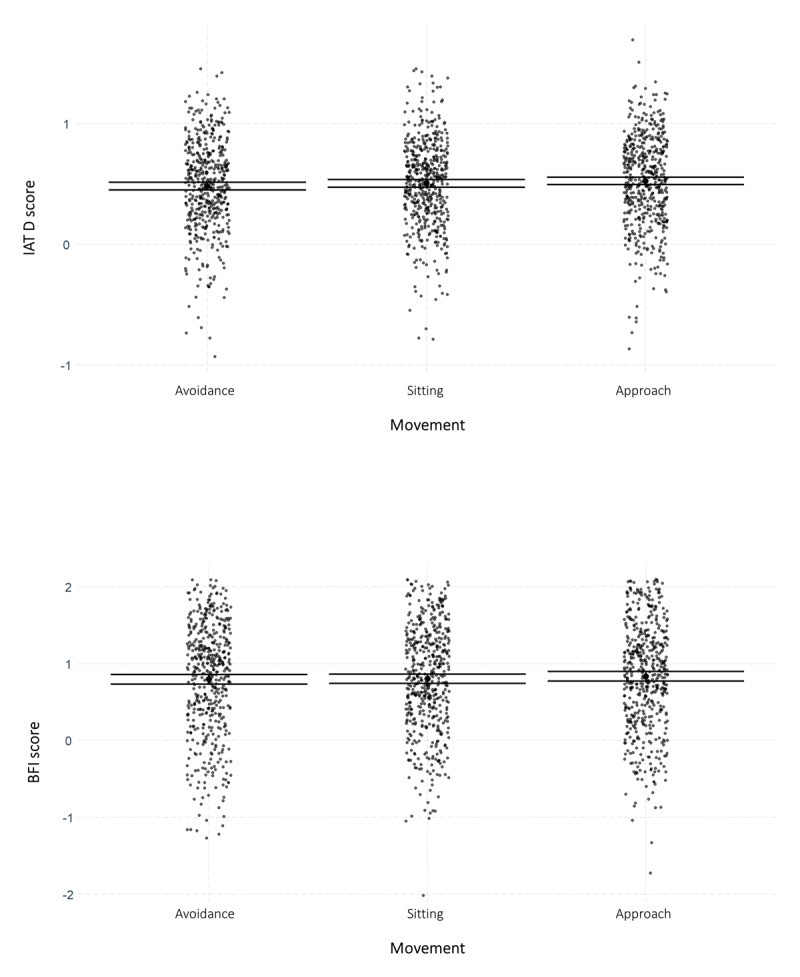
Self-evaluation proxies depending on movement in Experiment 3 (Error bars represent 95% confidence intervals).

For each participant, we calculated an IAT score (improved D-algorithm, Greenwald et al., 2003; R *IAT* package, version 0.3, Martin, 2016) such that higher D-scores indicated conscientiousness was more self-related than non-conscientiousness relative to others (split-half reliability computed on odd-even trials; *r* = .64). We computed a conscientiousness score by averaging responses on the BFI items (and after reversing-scoring the inverted items) such that higher scores reflected higher conscientiousness (α = *.88, M* = 0.81, *SD* = 0.70). We compared each distance-change condition with the control condition using a dummy coding (C1: approach = 1, control = 0, avoidance = 0; C2: approach = 0, control = 0, avoidance = 1). We ran a linear regression using the IAT score as the outcome and the dummy coding for the movement contingent on the dimension (C1; C2) as predictors. These analyses revealed that self-evaluation scores were not higher in the approach than in the control condition, nor were they lower in the avoidance than in the control condition, with all *BF*s indicating anecdotal evidence in favour of the null hypothesis (see Table 1 and Figure 3).^15^

### Discussion

We again failed to obtain an effect of approach on the conscientiousness dimension, both on a standard IAT and on self-reports, and this despite the fact that we increased the IAT’s reliability and statistical power.

Here, we added an avoidance condition to: 1) verify whether we replicated the approach (vs. avoidance) self-assimilative effect observed in the literature, 2) identify which of the conditions drives the effect, and 3) oppose two theoretical explanations. As the effect was not replicated, we cannot provide information for the two latter points.

## General Discussion

We proposed an innovative methodology designed to isolate the causal effect of specific behaviours on self-evaluation. We adapted the MT to manipulate distance variations between the on-screen self-representation (figure) and social information, while circumventing the shortcomings of previous paradigms. We first relied on this task to isolate the specific effect of approach and later, by adding avoidance, to illustrate how it can be used to evaluate competing theories. Results of three experiments failed to reveal the predicted self-assimilative effect of approach. *BF*s indicated no evidence or anecdotal evidence in favour of the null hypothesis.^16^

Our failure to observe an effect, despite increased experimental control, may suggest that the previously reported approach–avoidance training effect is mainly driven by a contrast effect in the avoidance condition. Nevertheless, Experiment 3 included an avoidance condition but found no effects for either approach or avoidance. It is therefore possible that some factor prevented us from observing the effect in the present setting.

One possibility is that, once confounds are controlled, the self-assimilative effect of approach is smaller than previously assumed. Thus, even our well-powered Experiment 3 may not have been optimal. If this is the case, one may question the relevance of investigating this effect. However, while typical experiments involve a concentrated training, in everyday life, individuals approach social information in a more distributed manner. In the learning literature, this distributed training is considered to be more effective (Gerbier & Toppino, 2015). Thus, even small effects might be consequential in the long run (Funder & Ozer, 2019).

A second possibility is that the effect of approach on self-evaluation may depend on individuals’ baseline level on the targeted dimension. Indeed, research suggests that approach fosters self-assimilation with categories with which individuals do not initially identify (Batailler et al., 2021; Phills et al., 2011). Some studies find the effect regardless of initial self-evaluation (but controlling for it, Boissicat et al., 2022), while others suggest it may be a moderating factor (Kawakami et al., 2008). It is thus possible that, if individuals evaluate themselves highly along the to-be-assimilated dimension, the effect may be constrained.

Another possibility concerns the used proxies of self-evaluation. Indeed, the RT-based measurements used here (i.e., LDT, IAT) may be questioned because they measure word recognition or categorisation speed, not evaluation per se. They may also be criticised for their low reliability. At first glance, this may explain the failed replication due to great variability in measurements. However, this explanation seems unlikely, as we also failed to obtain the effect on measurements directly targeting self-evaluation.

A final possibility is that our intention to implement stringent controls in the MT may have weakened the manipulation of interest or altered variables that constituted boundary conditions for the self-assimilative effect of approach. For instance, the MT involved grammatical categorisation rather than categorisation based on the dimension being evaluated. This was intended to reduce demand effects. However, because movement–stimulus contingency awareness could condition approach–avoidance training effects (Van Dessel et al., 2016), doing so may also have weakened the manipulation of interest. Moreover, to maximise experimental control, we avoided explicit approach–avoidance instructions that may be sufficient to create the effect (Van Dessel et al., 2017) and removed first-person visual flow, an undoubtedly important feature (Rougier et al., 2018). We instead capitalised on distance variations between a third-person self-representation (a figure labelled with participants’ first name) and the stimuli. This may have hindered interpreting behaviours as a distance reduction between the self and the stimuli. A pretest and the final item included in Experiment 3 suggest correct interpretations of the movements. However, in the absence of visual flow or explicit instructions, individuals may not construe the task as involving self-initiated approach and may not interpret the figure’s movement as their own. Research on approach–avoidance training and stimulus evaluation suggest that merely interpreting a movement as approach–avoidance can affect stimulus evaluation, whether participants perform the action or just observe it (Van Dessel et al., 2018; but see Hütter & Genschow, 2020). Effects of behaviours on self-evaluation, however, may require greater self-involvement before changes in the distance between self-representation and stimuli are reflected at a more conceptual level. Relatedly, recent research has shown that autonomy potentiates the evaluative influence of approach–avoidance (Niese & Hütter, 2025). Finally, given the context-dependency of approach–avoidance, our controlled settings may have mitigated the interpretation of approach as similarity. The contextual meaning of approach may constrain its self-assimilative effect. Overall, our decisions to increase control—and their consequences for the awareness of movement-dimension contingency, movement interpretation, and meaning—combined with indirect measurements of the dimension may have ultimately resulted in too strong a test of the effect that rendered it difficult to detect. Future research should establish whether these variables define boundary conditions of self-evaluative effects of behaviour and consequently, constitute limitations of the present study.

To conclude, we argue that isolating specific behavioural effects on self-construal is crucial to a better understanding of their underlying mechanisms and theory testing. We recommend carefully considering the control condition and proposed a task designed to single out the effect, although not immune to criticism. Crucially, we do not replicate the effect of either approach or avoidance. The present contribution points out to some crucially overlooked aspects of previous literature and emphasises once again the necessity of replication and the adoption of a cumulative view of science.

## Data Availability

Materials, data, and analytic (R) scripts are available at https://doi.org/10.17605/OSF.IO/CFRJ2.

## Footnotes

Despite the existence of possible moderators (e.g., information extremity or context), the literature supports the generality of the assimilation effect.

We reported the 90% CIs because the underlying test is an F-test and therefore a one-sided test. As reported by Lakens (2013) while the 90% CI always excludes 0 when the F-test is statistically significant, this is not the case for the 95% one.

The use of a single category and recoding-free procedure addresses some criticisms of the standard IAT (Teige-Mocigemba et al., 2010). Unipolar IATs enable researchers to deal with self-ambivalence (i.e., the co-presence of negative and positive self-evaluation) that characterises individuals with low self-esteem (Riketta & Ziegler, 2006; Houben et al., 2010).

Boissicat et al. (2022) obtained an effect size *d =* 0.29 (η^2^ = .02). However, the current work was conducted prior to the availability of these results.

In both experiments, excluded participants were distributed roughly evenly across conditions and the patterns of results remain the same with or without excluding participants.

See Supplementary Material, 1 for procedural details.

Suppression of latencies that were below the first quartile minus 1.5 times the interquartile range or above the third quartile plus 1.5 times the interquartile range.

A post-test (*N* = 37) revealed that two ‘neutral’ traits were rated positively (see Data and R scripts for Experiment 1 in OSF: I_Approach_Therefore_I_Am_Exp1_Analysis_Comment.R). We included them in score computation but weighted each trait score by its post-test rating.

This index was computed for the sake of comparability with Experiment 1.

Two participants were excluded due to studentised deleted residuals above four in the signature size change analysis.

A pretest revealed that the average interpretation for the sitting behaviour is equivalent to the ‘*Neither approach nor avoidance*’ interpretation (see Movement pre-test for Pre-registered Experiment 3 in OSF).

Although solutions exist to circumvent IAT limitations (e.g., the application of a multiple processing tree model), these generally implies modifying the standard IAT structure used in the literature.

One might argue that associative theories can predict both self-assimilation and self-contrast through the formation of an association between the acquired meaning of approach/avoidance in terms of similarity/difference, the self, and encountered information. However, this is not the most straightforward prediction of the self-anchoring account.

See Supplementary Material, 4, for details.

Exploratory analyses, detailed in Supplementary Material, 5, showed that the self-assimilation effect did not emerge when comparing approach to avoidance as done in past research. In addition, although participants correctly interpreted the figure’s movement, this interpretation did not influence self-evaluation.

Although conclusions must be tempered due to the limited number of included effects, we conducted a mini-meta-analysis across experiments. Results were not significant (see Supplementary Material, 6).

## References

[1] Batailler, C., Muller, D., Nurra, C., Rougier, M., & Trouilloud, D. (2021). Math approach training changes implicit identification with math: A close preregistered replication. Journal of Experimental Social Psychology, 92, Article 104059. 10.1016/j.jesp.2020.104059

[2] Boies, K., Lee, K., Ashton, M. C., Pascal, S., & Nicol, A. A. M. (2001). The structure of the French personality lexicon. European Journal of Personality, 15(4), 277–295. 10.1002/per.411

[3] Boissicat, N., Fayant, M. P., Nurra, C., & Muller, D. (2022). Social comparison in the classroom: Priming approach/avoidance changes the impact of social comparison on self-evaluation and performance. British Journal of Educational Psychology, 92(2), 594–609. 10.1111/bjep.12466 34729763

[4] Bonin, P., Méot, A., Aubert, L., Malardier, N., Niedenthal, P., & Capelle-Toczek, M.-C. (2003). Normes de concrétude, de valeur d’imagerie, de fréquence subjective et de valence émotionnelle pour 866 mots. L’Année Psychologique, 103(4), 655–694. 10.3406/psy.2003.29658

[5] Brendl, C. M., & Higgins, E. T. (1996). Principles of judging valence: What makes events positive or negative? In M. P. Zanna (Ed.), Advances in Experimental Social Psychology (Vol. 28, pp. 95–160). Academic Press. 10.1016/S0065-2601(08)60237-3

[6] Corneille, O., & Béna, J. (2023). Instruction-based replication studies raise challenging questions for psychological science. Collabra: Psychology, 9(1). 10.1525/collabra.82234

[7] Corr, P. J., & McNaughton, N. (2012). Neuroscience and approach/avoidance personality traits: A two stage (valuation–motivation) approach. Neuroscience and Biobehavioral Reviews, 36(10), 2339–2354. 10.1016/j.neubiorev.2012.09.013 23041073

[8] De Houwer, J., Crombez, G., Baeyens, F., & Hermans, D. (2001). On the generality of the affective Simon effect. Cognition and Emotion, 15(2), 189–206. 10.1080/02699930125883

[9] Dijksterhuis, A., Spears, R., Postmes, T., Stapel, D., Koomen, W., Knippenberg, A. v., & Scheepers, D. (1998). Seeing one thing and doing another: Contrast effects in automatic behaviour. Journal of Personality and Social Psychology, 75(4), 862–871. 10.1037/0022-3514.75.4.862

[10] Elliot, A. J. (2006). The hierarchical model of approach-avoidance motivation. Motivation and Emotion, 30(2), 111–116. 10.1007/s11031-006-9028-7

[11] Fayant, M. P., Muller, D., Nurra, C., Alexopoulos, T., & Palluel-Germain, R. (2011). Moving forward is not only a metaphor: Approach and avoidance lead to self-evaluative assimilation and contrast. Journal of Experimental Social Psychology, 47(1), 241–245. 10.1016/j.jesp.2010.07.013

[12] Franconeri, S. L., & Simons, D. J. (2003). Moving and looming stimuli capture attention. Perception & Psychophysics, 65(7), 999–1010. 10.3758/BF03194829 14674628

[13] Funder, D. C., & Ozer, D. J. (2019). Evaluating effect size in psychological research: Sense and nonsense. Advances in Methods and Practices in Psychological Science, 2(2), 156–168. 10.1177/2515245919847202

[14] Gallagher, S. (2000). Philosophical conceptions of the self: implications for cognitive science. Trends in Cognitive Sciences, 4(1), 14–21. 10.1016/S1364-6613(99)01417-5 10637618

[15] Gawronski, B., & Bodenhausen, G. V. (2011). The associative-propositional evaluation model: Theory, evidence, and open questions. In J. M. Olson & M. P. Zanna (Eds.), Advances in Experimental Social Psychology (Vol. 44, pp. 59–127). Academic Press. 10.1016/B978-0-12-385522-0.00002-0

[16] Gawronski, B., Bodenhausen, G. V., & Becker, A. P. (2007). I like it, because I like myself: Associative self-anchoring and post-decisional change of implicit evaluations. Journal of Experimental Social Psychology, 43(2), 221–232. 10.1016/j.jesp.2006.04.001

[17] Gerbier, E., & Toppino, T. C. (2015). The effect of distributed practice: Neuroscience, cognition, and education. Trends in Neuroscience and Education, 4(3), 49–59. 10.1016/j.tine.2015.01.001

[18] Greenwald, A. G., Brendl, M., Cai, H., Cvencek, D., Dovidio, J. F., Friese, M., Hahn, A., Hehman, E., Hofmann, W., Hughes, S., Hussey, I., Jordan, C., Kirby, T. A., Lai, C. K., Lang, J. W. B., Lindgren, K. P., Maison, D., Ostafin, B. D., Rae, J. R., … Wiers, R. W. (2022). Best research practices for using the Implicit Association Test. Behavior Research Methods, 54(3), 1161–1180. 10.3758/s13428-021-01624-3 34519017 PMC9170636

[19] Greenwald, A. G., McGhee, D. E., & Schwartz, J. L. K. (1998). Measuring individual differences in implicit cognition: The implicit Association Test. Journal of Personality and Social Psychology, 74(6), 1464–1480. 10.1037/0022-3514.74.6.1464 9654756

[20] Greenwald, A. G., Nosek, B. A., & Banaji, M. R. (2003). Understanding and using the Implicit Association Test: I. An improved scoring algorithm. Journal of Personality and Social Psychology, 85(2), 197–216. 10.1037/0022-3514.85.2.197 12916565

[21] Haynes, A., Kemps, E., & Moffitt, R. (2016). Is cake more appealing in the afternoon? Time of day is associated with control over automatic positive responses to unhealthy food. Food Quality and Preference, 54, 67–74. 10.1016/j.foodqual.2016.07.004

[22] Hommel, B., Müsseler, J., Aschersleben, G., & Prinz, W. (2001). The theory of event coding (TEC): A framework for perception and action. Behavioral and Brain Sciences, 24(5), 849–878. 10.1017/s0140525x01000103 12239891

[23] Houben, K., Nosek, B. A., & Wiers, R. W. (2010). Seeing the forest through the trees: A comparison of different IAT variants measuring implicit alcohol associations. Drug and Alcohol Dependence, 106(2–3), 204–211. 10.1016/j.drugalcdep.2009.08.016 19781863

[24] Hughes, S., Mattavelli, S., & De Houwer, J. (2018). Examining the impact of distance as a contextual cue in evaluative conditioning. PLoS ONE, 13(10), Article e0204855. 10.1371/journal.pone.0204855 PMC6171866 30286125

[25] Hütter, M., & Genschow, O. (2020). What is learned in approach-avoidance tasks? On the scope and generalizability of approach-avoidance effects. Journal of Experimental Psychology: General, 149(8), 1460–1476. 10.1037/xge0000728 31916835

[26] Judd, C. M., McClelland, G. H., & Ryan, C. S. (2009). Data analysis: A model comparison approach (2nd ed.). Routledge/Taylor & Francis Group.

[27] Kawakami, K., Steele, J. R., Cifa, C., Phills, C. E., & Dovidio, J. F. (2008). Approaching math increases math = me and math = pleasant. Journal of Experimental Social Psychology, 44, 818–825. 10.1016/j.jesp.2007.07.009

[28] Krieglmeyer, R., & Deutsch, R. (2010). Comparing measures of approach-avoidance behaviour: The manikin task vs. two versions of the joystick task. Cognition and Emotion, 24(5), 810–828. 10.1080/02699930903047298

[29] Lakens, D. (2013). Calculating and reporting effect sizes to facilitate cumulative science: a practical primer for t-tests and ANOVAs. Frontiers in Psychology, 4, Article 863. 10.3389/fpsyg.2013.00863 PMC3840331 24324449

[30] Lebens, H., Roefs, A., Martijn, C., Houben, K., Nederkoorn, C., & Jansen, A. (2011). Making implicit measures of associations with snack foods more negative through evaluative conditioning. Eating Behaviors, 12(4), 249–253. 10.1016/j.eatbeh.2011.07.001 22051355

[31] Lee, M. D., & Wagenmakers, E.-J. (2013). Bayesian cognitive modeling: A practical course. Cambridge University Press. 10.1017/CBO9781139087759

[32] Lignier, B., Petot, J.-M., Canada, B., De Oliveira, P., Nicolas, M., Courtois, R., John, O. P., Plaisant, O., & Soto, C. (2023). Factor structure, psychometric properties, and validity of the big five inventory-2 facets: Evidence from the French adaptation (BFI-2-FR). Current Psychology: A Journal for Diverse Perspectives on Diverse Psychological Issues, 42, 26099–26114. 10.1007/s12144-022-03648-0

[33] Markus, H., & Wurf, E. (1987). The dynamic self-concept: A social psychological perspective. Annual Review of Psychology, 38, 299–337. 10.1146/annurev.ps.38.020187.001503

[34] Martin, D. (2016). Cleaning and Visualizing Implicit Association Test (IAT) Data. https://download.nust.na/pub3/cran/web/packages/IAT/IAT.pdf

[35] Morey, R. D., Rouder, J. N., Jamil, T., Urbanek, S., Forner, Karl., & Ly, A. (2024). BayesFactor:Computation of Bayes Factors for Common Designs. https://CRAN.R-project.org/package=BayesFactor

[36] Morf, C. C., & Mischel, W. (2012). The self as a psycho-social dynamic processing system: Toward a converging science of selfhood. In M. R. Leary & J. P. Tangney (Eds.), Handbook of Self and Identity (2nd ed., pp. 21–49). The Guilford Press.

[37] Niese, Z. A., & Hütter, M. (2025). Choosing to avoid: The evaluative impact of autonomous selection of approach and avoidance behaviors. Journal of Experimental Psychology: Learning, Memory, and Cognition, 52(4), 505–521. 10.1037/xlm0001507 40569729

[38] Nussinson, R., Seibt, B., Häfner, M., & Strack, F. (2010). Come a bit closer: approach motor actions lead to feeling similar and behavioral assimilation. Social Cognition, 28(1), 40–58. 10.1521/soco.2010.28.1.40

[39] Phills, C. E., Kawakami, K., Tabi, E., Nadolny, D., & Inzlicht, M. (2011). Mind the gap: Increasing associations between the self and Blacks with approach behaviors. Journal of Personality and Social Psychology, 100(2), 197–210. 10.1037/a0022159 21299313

[40] Ric, F., Alexopoulos, T., Muller, D., & Aubé, B. (2013). Emotional norms for 524 French personality trait words. Behavior Research Methods, 45(2), 414–421. 10.3758/s13428-012-0276-z 23263927

[41] Riketta, M., & Ziegler, R. (2006). Self-ambivalence and self-esteem. Current Psychology, 25, 192–211. 10.1007/s12144-006-1003-7

[42] Rougier, M., Muller, D., Ric, F., Alexopoulos, T., Batailler, C., Smeding, A., & Aubé, B. (2018). A new look at sensorimotor aspects in approach/avoidance tendencies: The role of visual whole-body movement information. Journal of Experimental Social Psychology, 76, 42–53. 10.1016/j.jesp.2017.12.004

[43] Sanitioso, R. B., & Niedenthal, P. M. (2006). Motivated self-perception and perceived ease in recall of autobiographical memories. Self and Identity, 5(1), 73–84. 10.1080/15298860500386848

[44] Sedikides, C., & Strube, M. J. (1997). Self evaluation: To thine own self be good, to thine own self be sure, to thine own self be true, and to thine own self be better. In M. P. Zanna (Ed.), Advances in Experimental Social Psychology (Vol. 29, pp. 209–269). Academic Press. 10.1016/S0065-2601(08)60018-0

[45] Seibt, B., Neumann, R., Nussinson, R., & Strack, F. (2008). Movement direction or change in distance? Self- and object-related approach-avoidance motions. Journal of Experimental Social Psychology, 44(3), 713–720. 10.1016/j.jesp.2007.04.013

[46] Simmons, J. P., Nelson, L. D., & Simonsohn, U. (2013, 17–19 January). Life after p-hacking. Meeting of the Society for Personality and Social Psychology, New Orleans, LA, 10.2139/ssrn.2205186

[47] Solzbacher, J., Koenig, P., & Walter, S. (2025). Embodying “good” and “bad”: The emergent bodily meaning of approach- and avoidance-behavior. Philosophical Psychology, 39(4), 1529–1560. 10.1080/09515089.2025.2466646

[48] Soto, C. J., & John, O. P. (2017). The next Big Five Inventory (BFI-2): Developing and assessing a hierarchical model with 15 facets to enhance bandwidth, fidelity, and predictive power. Journal of Personality and Social Psychology, 113(1), 117–143. 10.1037/pspp0000096 27055049

[49] Spalding, L. R., & Hardin, C. D. (1999). Unconscious unease and self-handicapping: Behavioral consequences of individual differences in implicit and explicit self-esteem. Psychological Science, 10(6), 535–539. 10.1111/1467-9280.00202

[50] Suls, J., & Wheeler, L. (2007). Psychological magnetism: A brief history of assimilation and contrast in psychology. In D. A. Stapel & J. Suls (Eds.), Assimilation and contrast in social psychology (pp. 9–44). Psychology Press.

[51] Teige-Mocigemba, S., Klauer, K. C., & Sherman, J. W. (2010). A practical guide to the Implicit Association Test and related tasks. In B. Gawronski & B. K. Payne (Eds.), Handbook of implicit social cognition: Measurement, theory, and applications (pp. 117–139). The Guilford Press.

[52] Tukey, J. W. (1977). Exploratory Data Analysis. Addison-Wesley.

[53] van Dantzig, S., Pecher, D., & Zwaan, R. A. (2008). Approach and avoidance as action effects. Quarterly Journal of Experimental Psychology, 61(9), 1298–1306. 10.1080/17470210802027987 19086189

[54] Van Dessel, P., De Houwer, J., & Gast, A. (2016). Approach–avoidance training effects are moderated by awareness of stimulus–action contingencies. Personality and Social Psychology Bulletin, 42(1), 81–93. 10.1177/0146167215615335 26567171

[55] Van Dessel, P., Eder, A. B., & Hughes, S. (2018). Mechanisms underlying effects of approach-avoidance training on stimulus evaluation. Journal of Experimental Psychology: Learning, Memory, and Cognition, 44(8), 1224–1241. 10.1037/xlm0000514 29648864

[56] Van Dessel, P., Gawronski, B., Smith, C. T., & De Houwer, J. (2017). Mechanisms underlying approach-avoidance instruction effects on implicit evaluation: Results of a preregistered adversarial collaboration. Journal of Experimental Social Psychology, 69, 23–32. 10.1016/j.jesp.2016.10.004

[57] Van Dessel, P., Hughes, S., & De Houwer, J. (2019). How do actions influence attitudes? An inferential account of the impact of action performance on stimulus evaluation. Personality and Social Psychology Review, 23(3), 267–284. 10.1177/1088868318795730 30229697

[58] Zweigenhaft, R. L., & Marlowe, D. (1973). Signature size: Studies in expressive movement. Journal of Consulting and Clinical Psychology, 40(3), 469–473. 10.1037/h0034503 4708124

